# High prevalence of side population in human cancer cell lines

**DOI:** 10.18632/oncoscience.300

**Published:** 2016-04-10

**Authors:** Maximilian Boesch, Alain G. Zeimet, Heidi Fiegl, Barbara Wolf, Julia Huber, Helmut Klocker, Guenther Gastl, Sieghart Sopper, Dominik Wolf

**Affiliations:** ^1^ Institute of Immunobiology, Kantonsspital St. Gallen, 9007 St. Gallen, Switzerland; ^2^ Internal Medicine V, Medical University of Innsbruck, 6020 Innsbruck, Austria; ^3^ Department of Gynecology and Obstetrics, Medical University of Innsbruck, 6020 Innsbruck, Austria; ^4^ Department of Urology, Medical University of Innsbruck, 6020 Innsbruck, Austria; ^5^ Medical Clinic III, University Clinic Bonn (UKB), 53127 Bonn, Germany

**Keywords:** side population, cancer cell line, stem cell, drug resistance, drug transporter

## Abstract

Cancer cell lines are essential platforms for performing cancer research on human cells. We here demonstrate that, across tumor entities, human cancer cell lines harbor minority populations of putative stem-like cells, molecularly defined by dye extrusion resulting in the side population phenotype. These findings establish a heterogeneous nature of human cancer cell lines and argue for their stem cell origin. This should be considered when interpreting research involving these model systems.

## REPORT

Cancer has long been regarded a clonal disease. While this view still applies to certain haematological malignancies, the majority of cancers, and in particular solid tumors, follow another structure and are characterized by tremendous cellular complexity[1]. This diversity is likely to be imposed by both genomic instability and cell-extrinsic factors, such as microenvironment and immune system. Moreover, the discovery of cancer stem cells (CSC) as an integral part of most tumors has further advanced the concept of intratumoral heterogeneity. Fundamentally, the CSC concept states that long-term tumor propagation, metastasis and relapse depend on small populations of phenotypically-distinct cancer cells endowed with unique functional properties[2]. Importantly, the CSC model also implies that specifically the CSC pool drives tumor heterogeneity, based on a key stem cell (SC) characteristic termed asymmetric division.

In search of a common signature for ovarian CSC, we recently analysed the expression of several CSC markers in various ovarian cancer cell lines (n=13). This screen revealed that of all markers tested (CD24, CD44, CD90, CD133, CD326, ALDH and side population (SP)), only the SP marker detecting dye-extrusion by drug transporters robustly identified minor populations in virtually all cell lines investigated (total prevalence: 12/13 cell lines) (Table 1)[3]. Functionally, these SP cells were clonogenic, readily engrafted tumors in immune-compromised mice and repopulated both the SP and the non-SP, hence meeting established criteria for CSC. Furthermore, using in-depth phenotyping by flow cytometry, we found evidence for tremendous cellular complexity of the cell lines, and this diversity was even found within the small SP compartments of stem-like cells[3].

**Table 1:** Flow cytometric screening for SP in various human cancer cell lines

Cell Line	SP	Size of SP	FTC-sensitive
**Adrenal Gland Cancer**			
NCI-H295R	yes	+++	no
**Breast Cancer**			
BT-20 (HTB-19)	yes	++	yes
Hs 578T (HTB-126)	yes	+	yes
MCF7 (HTB-22)	yes	+++	yes
SKBR3 (HTB-30)	yes	+	yes
**Colorectal Cancer**			
HCT-8 (HRT-18)	yes	++	no
HT-29 (HTB-38)	yes	++	yes
**Lung Cancer**			
A549	yes	+++	yes
Calu-6 (HTB-56)	no	——	——
Colo-699	yes	+	yes
**Ovarian Cancer**			
A2780	yes	+	yes
A2780V	yes	++	no
B2/92	yes	+	no
B16/92	yes	+	yes
B17/92	yes	+	yes
B74/93	yes	+	no
Caov-3 (HTB-75)	yes	+	yes
HOC-7	yes	+	yes
IGROV1	yes	+	no
MDAH 2774	yes	+	no
NIH:OVCAR-3 (HTB-161)	no	——	——
SKOV-3 (HTB-77)	yes	+	yes
SKOV-6	yes	++	no
**Prostate Cancer**			
DuCaP	no	——	——
DU 145 (HTB-81)	no	——	——
LAPC4	yes	+	no
LNCaP	no	——	——
PC-3	no	——	——

Cells were harvested at subconfluency and subjected to DyeCycle^TM^ Violet-based SP detection as described previously[3]. FTC sensitivity indicates ABCG2 expression by SP cells, whereas FTC insensitivity indicates expression of another drug transporter such as ABCB1. In the latter case, the SP compartment was functionally confirmed using blocking with verapamil. ‘+’, ‘++’ and ‘+++’ indicate a SP size of <1%, 1-10% and >10%, respectively. SP, side population; FTC, fumitremorgin C.

In view of the great heterogeneity that we found, the almost-mandatory presence of SP cells in the cell lines was striking. To elaborate whether this was an ovarian cancer-specific phenomenon, we performed SP detection also with human cancer cell lines derived from non-ovarian solid tumor entities including those of prostate (n=5), breast (n=4), lung (n=3), colorectum (n=2) and adrenal gland (n=1). These experiments revealed that the SP phenotype was again a robust marker, defining distinct populations in 4/4 cell lines of breast cancer, 2/3 cell lines of lung cancer, 2/2 cell lines of colorectal cancer, 1/1 cell line of adrenal gland cancer, and still 1/5 cell lines of prostate cancer (Table 1). Thus, of altogether 28 cell lines tested, SP cells were resolvable in 22 cell lines, yielding a formidable prevalence of 78.6%.

The high conservation of SP cells in human cancer cell lines indicates a functional role of this cell compartment. In ovarian and several other cancers for instance, we and others have shown that SP cells exhibit functional SC characteristics[3,4,5]. Thus, it is plausible that these cells are required for cell line propagation in long-term. Moreover, it is well-established that SP cells, due to high expression of drug transporters such as ABCG2 and ABCB1, have direct implications in multidrug resistance[6]. Hence, these cells might serve as ‘evolutionary backup’ to guarantee the survival of at least a sub-fraction of cells when exposure to cytotoxic compounds occurs. Drug-induced acquisition of ‘*de novo* resistance’ might therefore often be a misinterpretation of what actually happens, that is Darwinian selection of small, pre-existing cancer clones inherently showing resistance. In this respect, cancer cell lines are therefore very similar to antibiotic-treated bacterial cultures[7].

Altogether, we propose that phenotypic and functional heterogeneity is a hallmark of most human cancer cell lines that needs to be considered for the correct interpretation of findings obtained with these model systems. Especially for observations related to SC characteristics and drug resistance, the SP minority compartment might often be responsible. Henceforth, it will be important to elucidate whether SP cells can also be found at such high frequency in *ex vivo* tumor tissue and whether these primary cells exhibit functional SC traits as well. This could not only further our understanding of *in vivo* tumor evolution and biology, but could also provide rationale for developing treatment strategies against a specific SC pool conserved within the tremendous complexity of solid tumors.
